# Redo Laparoscopic Pyeloplasty in Infants and Children: Feasible and Effective

**DOI:** 10.3389/fped.2020.546741

**Published:** 2020-11-10

**Authors:** Hamdan Al-Hazmi, Matthieu Peycelon, Elisabeth Carricaburu, Gianantonio Manzoni, Khalid Fouda Neel, Liza Ali, Christine Grapin, Annabel Paye-Jaouen, Alaa El-Ghoneimi

**Affiliations:** ^1^Department of Pediatric Urology, Robert-Debré University Hospital, Assistance Publique - Hôpitaux de Paris (APHP), Paris, France; ^2^College of Medicine and King Saud University Medical City, King Saud University, Riyad, Saudi Arabia; ^3^National Reference Center of Rare Urinary Tract Malformations (MARVU), Paris, France; ^4^University of Paris, Paris, France; ^5^Department of Pediatric Urology Fondazione Cà Granda Ospedale Maggiore Policlinico, Milan, Italy

**Keywords:** redo laparoscopic pyeloplasty, uretero-pelvic junction obstruction, open pyeloplasty, minimally invasive surgical procedures, children

## Abstract

**Purpose:** To determine the feasibility and effectiveness of redo laparoscopic pyeloplasty among patients with failed previous pyeloplasty, specifically examining rates of success and complications.

**Materials and Methods:** We retrospectively reviewed the charts of all patients, who underwent redo laparoscopic pyeloplasty from 2006 to 2017. This included patients who underwent primary pyeloplasty at our institution and those referred for failures. Analysis included demographics, operative time, complications, length of hospital stay, complications, and success. Success was defined as improvement of symptoms and hydronephrosis and/or improvement in drainage demonstrated by diuretic renogram, especially in those with persistent hydronephrosis. Descriptive statistics are presented.

**Results:** We identified 22 patients who underwent redo laparoscopic pyeloplasty. All had Anderson-Hynes technique except two cases in which ureterocalicostomy was performed. Median (IQR) follow-up was 29 (2–120) months, median time between primary pyeloplasty and redo laparoscopic pyeloplasty was 12 (7–49) months. The median operative time was 200 (50–250) min, and median length of hospital stay was 3 (2–10) days. The procedure was feasible in all cases without conversion. During follow-up, all but two patients demonstrated an improvement in the symptoms and the degree of hydronephrosis. Ninety-one percent of patients experienced success and no major complications were noted.

**Conclusions:** Redo laparoscopic pyeloplasty is feasible and effective with a high success rate and low complication rate.

## Introduction

Secondary uretero-pelvic junction obstruction (UPJO) may occur following pyeloplasty in up to 11% of patients who may require redo surgical intervention (1). Redo surgical intervention (open, laparoscopic, or robotic) has been shown to be more effective than endourological procedures (JJ stent insertion, balloon dilatation, and endopyelotomy) (2, 3). Laparoscopic and robotic redo pyeloplasty are alternatives to redo open pyeloplasty (ROP), which have been reported with good success (2, 4, 5).

Redo laparoscopic pyeloplasty (RLP) offers a minimally invasive approach with the benefits of a shorter period of convalescence and decreased morbidity compared to open surgery; however, it requires advanced laparoscopic skills (6). Herein, we report our outcomes with redo laparoscopic pyeloplasty to determine the feasibility and effectiveness of this procedure in a relatively large case series. And our hypothesis was: do infants and children with persistent UPJO undergoing redo laparoscopic pyeloplasty have the same overall success rate in comparison to the ones reported in open redo pyeloplasty series?

## Materials and Methods

### Patient Selection and Study Design

After obtaining ethical board approval for conduct of the study, we retrospectively reviewed the charts of all patients who underwent laparoscopic pyeloplasty for secondary UPJO at a single institution, University Hospital of Robert-Debré, Paris, France, from December 2006 to October 2017. Inclusion criteria were all patients with persistent UPJO undergoing redo transperitoneal laparoscopic pyeloplasty at our institution regardless of if their primary pyeloplasty was performed at our institution or elsewhere. Exclusion criteria were: primary UPJO repair or any redo pyeloplasty performed by an open, retroperitoneal laparoscopic or robot-assisted approach.

### Variables and Outcome Measures

Variables collected from the reviewed charts included: patient sex, age at primary surgery and redo surgery; type of previous interventions and number of attempts to repair the UPJO; confirmation of persistent UPJO following initial surgery, both clinically and radiologically (renal ultrasound, dynamic renal scintigraphy (MAG-3) and/or magnetic resonance urography (MRU); indication for redo pyeloplasty; use of stents and drains; length of hospitalization; postoperative complications; need for readmission and subsequent procedures; and success rate.

Indications for redo laparoscopic pyeloplasty were persistent severe hydronephrosis (defined as (1) AP diameter > 30 mm or (2) AP diameter > 15 mm and flank pain or (3) AP diameter > 15 mm and other US criteria (calyceal dilation, thin parenchyma)] associated with at least one of the following: repeated febrile urinary tract infection (UTI) documented by positive urine culture, flank pain, and persistence obstruction on retro or ante grade imaging (retrograde pyelography, renal scintigraphy, MRU). Surgical complications were classified according to the Clavien-Dindo classification (7). Febrile UTIs included both a fever and a urine cultures with >100,000 colony forming units.

Follow-up evaluation was performed using renal ultrasound and dynamic renal scintigraphy. Success defined as improvement of symptoms (neither UTI, nor flank pain) and decrease of hydronephrosis, determined by the measurement of post-operative anteroposterior diameter (APD, in millimeters) and/or the absence of calyceal dilation. In patients with persistent hydronephrosis, an absence of obstruction on the drainage curve on functional imaging (defined as a *t*_1/2_ <20 min on nuclear scan) was used to define success. A single dedicated radiologist was not available to perform all follow-up imaging.

### Surgical Details

**Figure 1:**
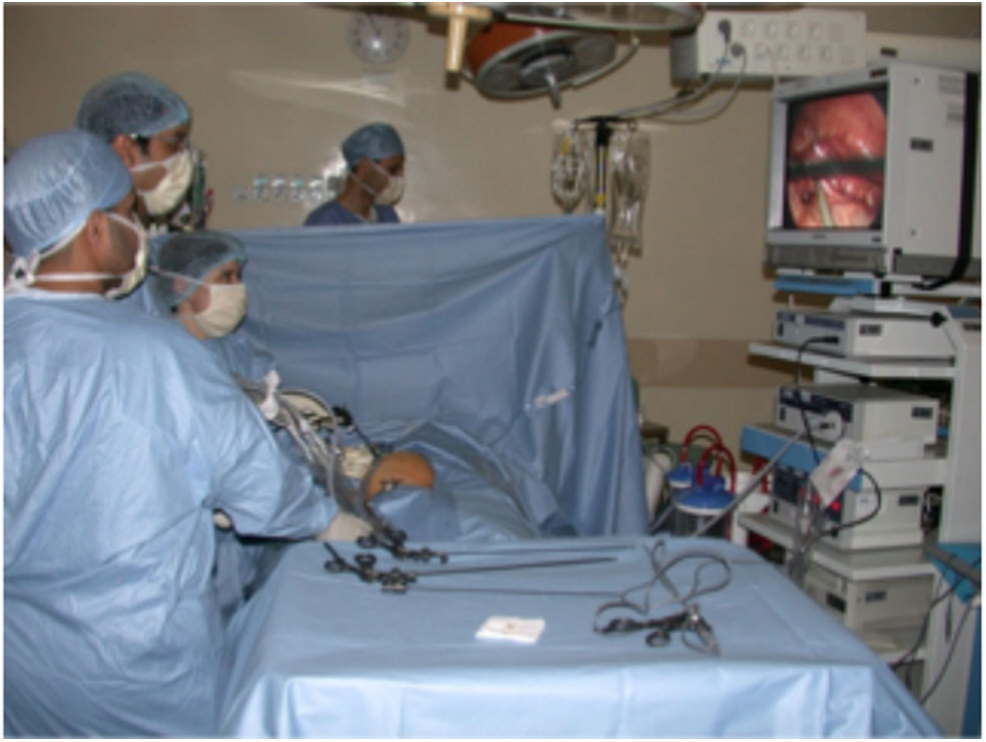
Position of the surgeon and all assistants.

**Figure 2:**
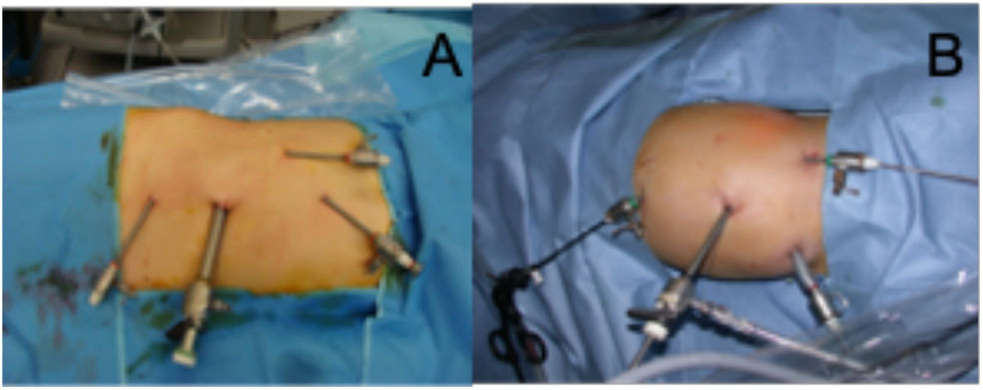
**(A)** Placement of the four trocars in older children: all in the midline; **(B)** Placement of the four trocars in young children.

The surgery was performed by staff pediatric urologists. A trans-peritoneal approach was used for all patients undergoing redo laparoscopic pyeloplasty. The patient was positioned in the supine position with an inflatable device under the flank of the operated side. The surgeon stood on the opposite side of the obstructed kidney (Figure 1), and all ports were inserted with the child in the supine position. Four ports were used for all patients (Figure 2), namely a 5-mm umbilical port by open access for the camera; and insufflation was maintained at 10 mm Hg. Then two 3-mm working ports were inserted under direct vision, one midway between the xiphoid process and umbilicus and the other midway between the symphysis pubis and the umbilicus, and the fourth accessory trocar, 3-mm, in the ipsilateral iliac fossa. The fourth was used to help to reduce the operative time for suction and exposition. There was some modification in the placing of trocars between young and older children (Figures 2A,B). A 45° lateral position was obtained by inflating the device. The colon was mobilized to expose the renal pelvis after removing all the adhesions until the UPJ was identified. The reason for the failure of previous surgery was identified. All patients underwent Anderson-Hynes dismembered pyeloplasty except two cases in which ureterocalicostomy was performed because the renal pelvis was intrarenal and difficult to identify due to severe fibrosis from the prior surgery, one of them had already failed redo robotic pyeloplasty elsewhere. Continuous suture was used for the anastomosis in all patients except one in whom interrupted suturing was required due to a thick-walled renal pelvis and signs of inflammation. We used a 5-0 Polyglactin suture for all patients. A JJ stent was placed in an antegrade fashion in all cases except one who had trouble in passing the JJ stent through the ureterovesical junction, so an externalized stent was placed instead (Multipurpose stent, BARD®, Salt Lake City, UT). A Foley catheter was placed and left until day 1 postoperatively.

All patients were followed-up clinically for pain or UTI, and radiologically by renal ultrasound (four times the 1st year, then twice the next year and finally once a year for 5 years). An isotopic renal scan or MRU was obtained in the setting of persistent severe hydronephrosis. The choice to use either an isotopic renal scan or a MRU was done on the functional imaging studies used for the preoperative evaluation.

Descriptive statistics were performed with SPSSV20 software (IBM SPSS Statistics, IBM Corp, Armonk, NY).

## Results

Twenty-two patients (four girls and 18 boys) underwent laparoscopic pyeloplasty for persistent UPJO during the study period. Thirteen patients (59.1%) were referred from an outside hospital after failed pyeloplasty. Median age at initial surgery was 8 months (IQR: 3–48). Surgery was performed on the right side in 10 patients and the left in 12 patients. Pre-operative micturating cysto-urethrogram (MCUG) was ordered in case of UTI after the first pyeloplasty (*N* = 4) and was normal in these selected patients except one patient with contralateral grade I vesicoureteral reflux that was observed. Previous surgical details are listed in Table 1.

**Table 1:** Previous surgical details (*N* = 22).

	**Patients (%)**
**Initial surgeries**	
Retroperitoneal laparoscopic pyeloplasty	3 (13.6)
Open dorsal lumbotomy	15 (68.1)
Open anterior subcostal incision	4 (18.2)
**Temporizing interventions**	
Nephrostomy tube	8 (36.4)
JJ stent	5 (22.7)
Endoscopic balloon dilatation	2 (9.1)
None	7 (31.2)

Median age at redo pyeloplasty was 22 months (IQR: 11–84 months), median weight at surgery was 10 kg (8–15 kg), and median time between primary and redo repair was 12 months (7–49 months). Patient details at time of redo laparoscopic pyeloplasty are shown in Table 2. Cause of failure of the primary repair was identified during laparoscopy as follows: adhesions around the UPJ area causing the obstruction (10 patients, 45.4%), stenotic UPJ area (seven patients, 31.8%), high anastomosis (anastomosis was not in the dependent area) (two patients, 9.1%), crossing vessels (one patient, post primary open repair, 4.5%), long segment stricture (one patient, 4.5%), and one patient had a twist of the anastomosis (4.5%) (Table 3). Preoperative and postoperative imaging features are reported in Table 4.

**Table 2:** Demographic and clinical data (*N* = 22).

	**Minimum**	**Maximum**	**Median**
Age at redo (months)	4.5	183	22
Weight (kg)	6	50	10
Operative time (min)	50	250	200
Hospital stay (days)	2	10	3
Follow-up duration (months)	2	120	29

**Table 3:** Side, intraoperative finding, procedure, and outcome (*N* = 22).

	**Number**	**Percentage (%)**
Symptoms	10	45.5
UTI	7	31.8
Pain	4	18.2
Asymptomatic	12	54.5
Obstruction side: Right/Left	10/12	45.5/54.5
**Intraoperative Cause of Failure**		
1. Adhesions causing obstruction	10	45.5
2. UPJ obstruction	7	31.8
3. Highly inserted ureter	2	9
4. Crossing vessels	1	4.5
5. Long segment stricture	1	4.5
6. Twist of the anastomosis	1	4.5
**Intraoperative Procedure:**		
1. Anderson-Hynes technique	20	90.9
2. Ureterocalicostomy	2	9.1
**Readmission**		
Yes	2	9.1
No	20	90.9
**Outcome**		
Success	20	90.9
Failure	2	9.1

**Table 4:** Preoperative and postoperative imaging features (N=22).

	**Preoperative evaluation**	**Postoperative evaluation**	***p*-value**
Anteroposterior diameter on renal ultrasound (mm) (median and IQR)	36 (34–50)	15 (9–45)	0.04
Functional imaging (*N*, %)			0.99
Renal scintigraphy	3 (13.6)	2 (9)	
MRU	19 (86.4)	10 (45.5)	
*t*_1/2_ (median and IQR)	40 (35–50)	14 (13.5–14.5)	
Split renal function on functional studies (%) (median and IQR)	32 (24–46)	33 (21–39)	0.79
Pyelography (*N*, %)			0.52
Antegrade	5 (22.7)	0 (0)	
Retrograde	8 (36.4)	2 (9)	

A JJ stent was used for all patients for a median duration of 2.5 months (IQR: 2–3 months). There was a single exception to this in the case of a patient in whom there was difficulty passing the JJ stent beyond the uretero-vesical junction, so an externalized ureteral catheter was used for 10 days.

Median operative time was calculated from the start of insufflation until exsufflation and was 200 min (IQR: 180–225 minutes). Median length of hospital stay was 3 days (IQR: 3–4.25 days). Two patients had a prolonged hospital stay: the first one kept admitted 10 days to await resolution of a urine leak from the anastomosis site. The other was readmitted on day 11 after surgery for pyelonephritis Intravenous antibiotics were injected at hospital for 6 days.

The procedure was feasible in all cases without conversion to open surgery. No major complications (Clavien ≥ III) were recorded.

Median follow-up duration was 29 months (IQR: 15–62 months). All patients were asymptomatic except one patient who presented with post-operative pain and pyelonephritis 11 days after surgery. Nineteen patients demonstrated an improvement in hydronephrosis. Three showed severe hydronephrosis with an obstructed curve on nuclear study. One patient had a wide dependent draining anastomosis on retrograde pyelography without obstruction and therefore was not considered a failure. The other two patients had obstruction confirmed on retrograde pyelography (RPG). In these patients, the kidney was palpable and on ultrasound exhibited worsening of hydronephrosis with the average APD increased from 33 mm to 51 mm and decreased renal function by renography from 25 to 7%. One underwent endoscopic balloon dilatation after the RPG and the second underwent a redo laparoscopic pyeloplasty. Both are doing well after their repeat intervention.

Overall success of redo laparoscopic pyeloplasty was 90.9%.

## Discussion

The first attempt of laparoscopic pyeloplasty for primary ureteropelvic junction obstruction was described for adults at the end of twentieth century in 1993 followed by reports for children in 1995 (8–10). Only one of these cases had secondary ureteropelvic junction obstruction after failure of open pyeloplasty (8). Since that time, the role of laparoscopic and robotic assisted pyeloplasty has evolved to take a more primary role in the management of primary UPJO, regardless of age or trans peritoneal vs. retroperitoneal approach (11–15). However, the gold standard for redo cases has general been considered an open pyeloplasty and thus redo laparoscopic pyeloplasty in children has not been widely applied. With the advent of improving minimally invasive techniques and increasing familiarity with these approaches, many have advocated using minimally invasive techniques in the redo setting. In a prospective, case–control study of open vs. laparoscopic pyeloplasty, 30 patients with UPJO were compared. This showed comparable results with the laparoscopic approach being associated with a decrease in hospital stay and complication rates when compared to children in the open cohort (16, 17).

Management options in failed pyeloplasty include JJ stent placement, balloon dilatation endopyelotomy, and redo surgery (2, 3). Lower success rates have been reported endoscopic procedures as compared to redo pyeloplasty, which is not surprising (2, 3, 18). However, Dy et al. reported that at least one endoscopic procedure was performed prior to definitive redo-pyeloplasty in 11% of children with failed pyeloplasty (1).

Performing a redo-UPJO is a challenging surgery. Despite encouraging outcomes achieved with both laparoscopy and robotics, success rates are likely to be lower than those obtained in the primary setting (19).

Redo laparoscopic pyeloplasty has well-established merits, including reduced morbidity, reduced hospital stays, and reduced pain compared to open pyeloplasty (19). However, this challenging technique must be performed by experienced surgeons due to the extensive scarring and fibrosis noted from the previous procedure (4). Basiri et al. evaluated the feasibility and effectiveness of RLP and found that 100% of children showed improved renal function after undergoing secondary UPJO treated by RLP, lending credence to its value over immediately attempting an open repair (20).

In our previously reported experience, primary laparoscopic pyeloplasty has a 98% success rate, which is higher than the 90.9% reported in the current study of redo laparoscopic pyeloplasties (21). Our current findings are similar to those reported by Abdel-Karim et al. (22). Similarly, Moscardi et al. had a 90% success rate of 11 redo laparoscopic pyeloplasty, and they showed no difference in the outcome between primary laparoscopic pyeloplasty and redo laparoscopic pyeloplasty in terms of operative time, complications, and success rate (23).

Redo laparoscopic pyeloplasty has been reported in adults and children usually using the transperitoneal approach but occasionally through a retroperitoneal approach (4, 24). Although the retroperitoneal approach is still our preference for primary cases, we have chosen the transperitoneal approach for secondary cases (21). This choice has been made to avoid dissecting through secondary adhesions in the retroperitoneal space, and to limit the dissection to the UPJ and proximal ureter. Interestingly, most of the cases that we report in the current study were performed in an open fashion for the primary surgery, and not all initially underwent a retroperitoneal approach for their initial surgery. It raises the question of whether a better option would be a retroperitoneal laparoscopic approach for a redo pyeloplasty if the patient originally underwent a transperitoneal approach.

Factors, such as young age at initial surgery (<6 months), missed anatomic findings at the first intervention (long ureteral segment narrowing or crossing vessels) and dry anastomosis (prolonged urinary diversion) have been associated with pyeloplasty failure (13, 25). The degree of adhesion and fibrosis is highly variable, which may be secondary to healing factors of the patients as well as the technical difficulty in the primary surgery, such as an incomplete or unfavorable position of the anastomosis between the renal pelvis and ureter or urinoma (26). Additionally, peri pelvic fibrosis, excessive scarring, and thermal energy, which can cause more tissue reactions and fibrosis can be associated with failures (27). These reasons support the findings we report in our cohort.

The long median time between primary and redo surgeries in our series is explained by the large (13 out of 22) number of patients referred to us from outside, which is the same observation noticed by Moscardi et al. (23). It would have been pertinent to examine factors associated with primary pyeloplasty failure, given the fact that over half of the patients were referrals, we did not feel that we could justify such an analysis using our data.

In our experience, two factors have been identified for the success of this procedure. First, the use of MRU as an anatomical and functional imaging studies during the preoperative management is a useful tool to assess the anatomy of the kidney and the renal pelvis, to measure the thickness of the parenchyma and to evaluate the split renal function. The first study from Perez-Brayfield et al. in 2003 concluded that dynamic contrast enhanced MRI provided equivalent information about renal function but superior information regarding morphology in a single study without ionizing radiation (28). A multi-institutional study in 2014 including 369 patients reported an equivalence of MRU to renal scintigraphy making substitution of MRU for RS acceptable (29). In our study, 19 (86.4%) patients were evaluated with a MRU. We strongly believe this imaging is better than a renal scintigraphy as it provides a better evaluation of the pelvis anatomy. Median split renal function of the operated kidney was 32 and 33% preoperatively and postoperatively, respectively (*p* > 0.05). However, functional studies were not unfortunately performed routinely after the redo surgery either in cases of preoperative evaluation showed asymmetrical function or remaining hydronephrosis. Secondly, the experience of our team in using minimally invasive surgery in our daily practice helps the laparoscopic approach to provide easily a global exposure of the pelvis and the ureter without the need to extensively dissect or mobilize the kidney (12, 21, 30–34). In selected cases with an extensive fibrosis of multi-operated renal pelvis, an alternative approach by ureterocalicostomy was deemed most appropriate.

There are multiple limitations worth discussing in the present study. First and foremost is the retrospective nature and small number of included patients. Furthermore, the fact that over half of the patients were referred to our institution makes it challenging to comment at all on how the initial surgical approach could have impacted the redo procedure that we report upon herein. The minority of patients underwent a laparoscopic pyeloplasty for their primary repair. However, as the primary goal of the study was to examine the feasibility and effectiveness of performing laparoscopic pyeloplasty in the redo setting, particularly in the setting of such a high proportion of prior open repairs, we feel that the limitations are acceptable so long as the reader is aware of them.

## Conclusion

Redo laparoscopic pyeloplasty is both a feasible and effective procedure for the management of failed primary pyeloplasty, regardless of whether the initial surgery was performed open or laparoscopic. Given the benefits of shorter hospitalization and reduced pain following any minimally invasive procedure, it should be strongly considered as an option for any pediatric patient presenting with a recurrent UPJ obstruction.

## Data Availability Statement

The raw data supporting the conclusions of this article will be made available by the authors, without undue reservation.

## Ethics Statement

The studies involving human participants were reviewed and approved by IRB and ethical board approval at our institution was obtained for this study. Written informed consent to participate in this study was provided by the participants' legal guardian/next of kin. Written informed consent was obtained from the minor(s)' legal guardian/next of kin for the publication of any potentially identifiable images or data included in this article.

## Author Contributions

HA-H, MP, EC, GM, CG, AP-J, and AE-G contributed conception and design of the study. HA-H, MP, EC, KN, LA, and AE-G organized the database. HA-H, LA, and MP performed the statistical analysis. HA-H wrote the first draft of the manuscript. MP, GM, AP-J, LA, and AE-G wrote sections of the manuscript. All authors contributed to manuscript revision, read, and approved the submitted version.

## Conflict of Interest

The authors declare that the research was conducted in the absence of any commercial or financial relationships that could be construed as a potential conflict of interest.

## Abbreviations

APD: Anteroposterior diameter
IQR: Interquartile
Kg: kilogram
MCUG: Micturating cysto-urethrogram
MRU: Magnetic resonance urography
OP: Open pyeloplasty
PP: Primary pyeloplasty
RLP: Redo laparoscopic pyeloplasty
ROP: Redo open pyeloplasty
UPJ: Uretero-pelvic junction
UPJO: Uretero-pelvic junction obstruction
UTI: Urinary tract infection.
